# Mood Symptoms, Suicide, and Associated Factors Among Jimma Community. A Cross-Sectional Study

**DOI:** 10.3389/fpsyt.2021.640575

**Published:** 2021-03-19

**Authors:** Yonas Tesfaye, Liyew Agenagnew, Susan Anand, Gudina Terefe Tucho, Zewdie Birhanu, Gutema Ahmed, Masrie Getnet, Kiddus Yitbarek

**Affiliations:** ^1^Department of Psychiatry, Jimma University, Jimma, Ethiopia; ^2^School of Nursing and Midwifery, Jimma University, Jimma, Ethiopia; ^3^Department of Environmental Health Sciences and Technology, Jimma University, Jimma, Ethiopia; ^4^Department of Health, Behavior, and Society, Jimma University, Jimma, Ethiopia; ^5^Department of Biostatistics and Epidemiology, Jimma University, Jimma, Ethiopia; ^6^Department of Health Policy and Management, Jimma University, Jimma, Ethiopia

**Keywords:** mood symptoms, anxiety symptoms, suicide, community, Jimma, Ethiopia

## Abstract

**Background:** The global burden of mental health problems is high and is predicted to rise. At present, mood symptoms are the foremost common psychological problems worldwide, yet little is known regarding their magnitude and associated factors in developing countries. Therefore, this study aimed to assess the magnitude and associated factors of anxiety, depressive, manic symptoms, and suicidal behavior among the rural Jimma community, Ethiopia.

**Methods:** A community-based quantitative cross-sectional survey was employed on 423 households selected through systematic random sampling. An adapted version of the Mini International Neuropsychiatric Interview tool was used for the structured face-to-face interview. The collected data were checked for completeness, coded, and inserted into Epi Data version 3.1 and exported to SPSS version 23 for analysis. Variables with *P* < g0.05 and odds ratio (OR) [95% confidence interval (CI)] on multivariate logistic regression analysis were considered as factors associated with the outcome variable.

**Results:** Overall, 185 (44.0%), 55 (13.1%), 44 (10.5%), and 23 (5.5%) of the respondents had anxiety, depressive, manic symptom, and suicide behavior, respectively. The odds of having anxiety symptoms were nearly 5 times higher among those who had perceived discrimination and racism experience compared to their counterpart [adjusted OR (AOR), 5.02; 95% CI, 1.90–13.26]. Likewise, recently bereaved participants had 4-fold higher odds of reporting depressive symptoms (AOR, 3.9; 95% CI, 1.4–10.4) than the non-bereaved ones. Furthermore, respondents who had depressive symptoms were almost four and a half times more likely to have manic symptoms compared to those who did not (AOR, 4.3; 95% CI, 1.71–11.02).

**Conclusion:** Anxiety, depressive, manic symptoms, and suicidal behavior were prevalent in the community and positively associated with multiple psychosocial factors. Implementing accessible and affordable community-based mental health services is recommended to mitigate the problems.

## Background

Mental health is vital to individual well-being, family bonding, and successful contributions to society. It is associated with the development of societies and countries (1, 2).

The global burden of mental health problems (mood, anxiety disorder, and suicide) is high and predicted to rise. At present, mood symptoms are the foremost common psychological problems worldwide, and it has been forecasted that unipolar depressive disorders will be the second leading cause of the burden of disease in 2030 (3–7).

Mental health problems are known to increase morbidity and mortality and are an important risk factor for adverse health outcomes. The social, economic, and health effects are extensive, where they are related to increased all-cause mortality, occupational disability, poor quality of life, and cardiovascular disease risk (8). Despite this, mental health is often ignored as public health priority (9).

Mental health problems such as mood disorders are associated with multiple factors such as gender, income (10), education level, socioeconomic conditions, medical illness (11, 12), age, substance use (13), stressful life events (14, 15), history of parental substance use (16), residence, marital status (17, 18), perceived racism and discrimination (19, 20), domestic violence (15), death of a close relative (21), birth order (22, 23), violence, migration, sexual abuse experience, life-threatening and physical injuries, difficulties with family relationships, and low emotional support at home during childhood (24, 25).

The World Health Organization (WHO) World Mental Health surveys show clearly that mental disorders are quite common in all the countries (26). However, developing countries, such as Ethiopia, are facing the impact of mental health problems while confronted with limited resources and inequities in access to mental health care (27).

More than three-quarters of people who have mental problems are residing in low- and middle-income countries (LMICs), with mental illness and substance use disorders presenting as an important cause of disease burden (28, 29). In many LMICs, there is typically a shortage of mental health professionals; with little or no multidisciplinary team and few regular drugs available, this can further worsen the impacts and burdens of the problems (30).

In Ethiopia, mental illness is the leading non-communicable disorder in terms of burden. Indeed, in a predominantly rural area of Ethiopia, mental illness comprised 11% of the total burden of disease. Severe mental illness is more often attributed to supernatural causes, rather than as a result of biomedical or psychosocial causes. The number of trained mental health professionals is inadequate for providing services to Ethiopians. There is only one dedicated psychiatric hospital in the entire country for more than 110 million population (31).

Evidence showed that in the rural context living conditions such as limited social and economic resources, stressful life events, poverty, and other demographic disadvantages pose a greater risk for mental health problems (32, 33). Also, various sociocultural factors such as deeply ingrained religious and inherited beliefs that all mental illnesses contribute to the existence and poor modern treatment for mental health problems in the country (34). In Ethiopia, in any ethnic or religious group, supernatural powers are given the attribute of controlling the well-being of the individual's mind. The traditional healing methods are used more by most people (35–37).

In Ethiopia, where undernutrition and preventable communicable diseases are very rampant, mental health problems, which are considered as non-fatal, are not given due consideration (38). Valid and inclusive epidemiological data on the magnitude and associated factor of mental health problems generated from community-based surveys have significant scientific and health policy implications (39). There are some research works documented in the literature regarding the magnitude and associated factors of anxiety, depressive, manic symptoms, and suicidal behavior among Ethiopians. However, in this study, variables such as migration history, perceived discrimination and racism experience, sexual abuse, and domestic violence were included. Furthermore, the studies on mental health problems are scarce in the sub-Saharan countries, and most studies were conducted in the cities. However, this study has tried to reveal the extent of mental health problems and associated factors in the neglected rural area of the country; this might help the local health planners and non-governmental organizations working in the area of mental health to investigate and design effective locally sound mental health interventions to avert the problems. Hence, this study aimed to assess the magnitude and associated factors of anxiety, depressive, manic symptoms, and suicidal behavior among the Jimma zone community, Ethiopia.

## Methods

### Study Setting

The study was carried in the Jimma zone, Seka Chekorsa district. Jimma zone is administratively divided into 20 districts and one town administration. The total population of the zone was 2,986,957 in 2017 (40). Seka Chekorsa district is located 20 km from Jimma town, and the district has 30 kebeles (the lowest administrative division in the area) with a total population of 208,096 (41). This district has one primary hospital, nine health centers, and 35 health posts. The study was conducted from March 1 to 22, 2020.

Ethiopia is one of the 213 countries that have registered 2019 coronavirus disease (COVID-19) cases since March 13, 2020. In Ethiopia, several cases and deaths are identified (42). The study data were collected in the first couple of weeks of the virus detection in the country.

### Sample Size Estimation

Single population proportion formula was used to obtain the desired sample size. We have assumed a 50% proportion of the magnitude of mood symptoms to get the maximum sample size, 95% confidence level, and 5% margin of error. Hence, *n* = (*z*α/2)^2^ *P*(1–*p*)/*d*^2^; hence, *n* = (1.96)^2^ × 0.5 (1–0.5)/(0.05)^2^ = 384. With the addition of a 10% contingency for non-response, the final sample size was 423.

### Study Design, Population, Sampling Technique, and Procedures

A community-based quantitative cross-sectional survey was carried out. First, Seka Chekorsa district was selected from a total of 20 districts in the zone through a simple random sampling lottery technique. Of the 30 kebeles in this district, nine were selected by lottery method based on the WHO sample size calculation guideline for the district health system (43). The number of sampled respondents from each kebele was determined by proportional allocation to the total number of households in each of the sampled kebeles. A systematic random sampling technique was used to select the study units, and periodic interval (*K*) was calculated using the formula *K* = *N*/*n*, whereby *N* is the total households in the selected kebeles (1,555), and *n* is the calculated sample size (423). Accordingly, every four households were included in the study. The first study unit was selected by lottery method between the first and fourth households. Finally, randomly selected household members 18 years or older in the selected household responded to the interview. The study participants were household members 18 years or older in the randomly selected households.

### Eligibility Criteria

All the study community members 18 years or older were included in the study. The study community members who were acutely or chronically ill, which makes him/her difficult to participate in the study, were excluded from the study.

### Measurements and Procedures

A face-to-face interviewer-administered structured questionnaire was used using an adapted version of the Mini International Neuropsychiatric Interview (M.I.N.I.). M.I.N.I. 5.0.0 was developed by Sheehan et al. and designed for assessing the major Axis I mental health problems in *Diagnostic and Statistical Manual of Mental Disorders, Fourth Edition* (*DSM-IV*) (researchers and clinicians working in non-profit or publicly owned settings can freely use for clinical and research use) (44). Validation and reliability of the tool have been done with good psychometric properties (45–47). The reliability, Cronbach α score, of the scales in this study was 0.76. This study has assessed the prevalence of *DSM-IV* criteria A symptoms of panic disorder (lifetime), social anxiety disorder (past month), generalized anxiety disorder (past 6 months), suicide characteristics (such as repeatedly consider hurting self, having a plan to kill self, repeatedly wish dead, and suicide attempt) (past month), a lifetime suicide attempt, major depression (current or 2 weeks), and manic episode (lifetime). The tool has also included questions to measure other family members with such symptom presentations. If the subject answers positive for any of the questions about symptoms included in M.I.N.I.'s anxiety, depressive, manic symptoms, and suicide module, it is considered to have the symptoms. For factors associated with the outcome variable, the questionnaire was developed after a thorough review of the literature that has been done on similar topics (32, 48–50); the presence or absence of these factors was measured using a structured question. Data were collected by 20 health extension workers after receiving training for 2 days on the components of the questionnaire, data recording, and the ethical principles of the data collection process. The English version of the questionnaire was translated into Afaan Oromo and back-translated to English to ensure its uniformity by blinded language experts. The translations were face validated by two independent external experts in the field. Moreover, the data collection tool was pilot tested on 5% of the population in another district to ensure its clarity and consistency. The pretest results showed the questionnaire was easily understandable, and the interview process was clear for the respondents. Afaan Oromo version of the questionnaire was used to obtain the desired data. Appropriate COVID-19 infection containment measures, which WHO recommended (keeping a 2-meter distance, wearing a face mask, and using alcohol-based hand sanitizers), were practiced during the data collection period.

### Data Organization and Statistical Analysis

The collected data were checked for completeness, given code, and inserted into Epi Data version 3.1 and exported to SPSS version 23 for analysis. Descriptive statistics were done to summarize the variables. The logistic regression analysis model was used to identify the factor associated with the outcome variable. First, bivariate logistic regression analysis was done, and variables with *p* < 0.25 were selected as candidate variables for multivariate logistic regression analysis. After the model was tested for multicollinearity and Hosmer–Lemeshow test of model fitness, the final multivariate logistic regression analysis was carried out. Finally, variables with *p* < 0.05 and 95% confidence interval and odds ratio (OR) were considered as factors associated with the outcome variable.

### Ethical Consideration

Ethical approval was obtained from the Institutional Review Board (IRB) of Jimma University (IHRPGD/584/2019). Additionally, a support letter was found from Oromia Regional Health Bureau, Jimma Zone Health Bureau, and a subsequent support letter was obtained from the Seka Chekorsa district health office before the commencement of data collection. Respondents were informed of the study objectives and were assured of the anonymity of their participation. Participation in the study was voluntary, and written informed consent was taken from the respondents. Respondents who had anxiety, depressive and manic symptoms, and suicidal behavior were advised to visit the nearby health facility for further mental health evaluation and management.

## Results

### Sociodemographic Characteristics

A total of 420 study participants were interviewed successfully, giving a response rate of 99.3%. Three respondents were not willing to participate in the study. The mean age of the respondents was 37.2 years (SD, ±11.9 years) with a range of 18 to 80 years. The majority of the study respondents were females [230 (54.8%)], married [345 (82.1%)], Oromo ethnic group [395 (94%)], Muslim [384 (91.4%)], and unable to read and write [168 (40.0%)]. The mean monthly income was 1,562.5 Ethiopian Birr (ETB) (SD, ±2,769.8) (approximately US $48.00) (Table 1).

**Table 1:** Sociodemographic characteristics of respondents at Jimma Zone, Seka Chekorsa district, Southwest Ethiopia, March 2020.

**Variables**	**Characteristics**	**Frequency**	**Percentage**
Sex	Male	190	45.2
	Female	230	54.8
Age	37.2 (SD, ±11.9)		
	Range 18–80 years		
Residence	Urban	167	39.8
	Rural	253	60.2
Birth order	First	183	43.6
	Second	102	24.3
	Third	60	14.3
	Fourth or more	75	17.9
Ethnicity	Oromo	395	94.0
	Amhara	12	2.9
	Others^*^	13	3.1
Religion	Muslim	384	91.4
	Orthodox	24	5.7
	Others^†^	12	2.8
Monthly income	1,562.5 (SD, ±2,769.8)		
Educational status	Unable to read and write	168	40.0
	Able to read and write	81	19.3
	Primary school (grades 1–8)	112	26.7
	Secondary school (grades 9–12)	49	11.7
	Diploma	6	1.4
	Degree and above	4	1.0
Marital status	Single	30	7.1
	Married	345	82.1
	Divorced	12	2.9
	Widowed	33	7.9
Occupational status	Farmer	219	52.1
	Merchant	61	14.5
	Daily laborer	18	4.3
	Housewife	100	23.8
	Employed	22	5.3

* *Other ethnicities include Kefa, yem, Dawro, Gurage, and Silte*.

† *Other religions include Protestant, Catholic, and Wakefeta*.

### Anxiety Symptoms

Twenty-eight participants (6.7%) had spells or attacks of sudden anxiety, frightened, uncomfortable, or uneasy, even in situations where most people would not feel that way on more than one occasion. Similarly, about 71 (16.9%) respondents were fearful or embarrassed by being watched including things such as speaking in public, eating in public or with others, writing while someone watches, or being in social situations in the past month. Nearly one-quarter [101 (24.0%)] of the participants were worried excessively or had been anxious about several things over the past 6 months. Overall, 185 (44.0%) seemed to have anxiety symptoms (Table 2).

**Table 2:** Anxiety symptoms characteristics of the respondents and their family at Jimma Zone, Seka Chekorsa district, Southwest Ethiopia, March 2020.

**Variable**	**Characteristics**	**Frequency**	**Percentage**
On more than one occasion; had spells or attacks; suddenly felt anxious, frightened, uncomfortable, or uneasy, even in situations where most people would not feel that way	Yes	28	6.7
	No	392	93.3
Other family members having these	Yes	18	4.3
	No	402	95.7
No. of family members having these	One	15	83.3
	Two	3	16.7
Experience of spells surge to a peak within 10 min of starting?	Yes	19	67.8
	No	9	32.2
At any time in the past, those spells or attacks come on unexpectedly or occur in an unpredictable or unprovoked manner	Yes	26	92.8
	No	2	7.2
**Ever had one such attack followed by a month or more of**			
Persistent concern about having another attack	Yes	22	78.6
	No	6	21.4
Worries about the consequences of the attack	Yes	23	82.1
	No	5	7.9
The significant change in your behavior because of the attacks	Yes	18	64.3
	No	10	35.7
Other family member having these	Yes	49	11.7
	No	371	88.3
No. of family members having these	One	45	91.8
	Two	2	4.1
	Three	2	4.1
**In the past month, including things like speaking in public, eating in public or with others, writing while someone watches, or being in social situations**			
Fearful or embarrassed about being watched	Yes	68	16.2
	No	352	83.8
Being the focus of attention	Yes	71	16.9
	No	349	83.1
Fearful of being humiliated	Yes	43	10.2
	No	377	89.8
Other family member having these	Yes	22	5.2
	No	398	94.8
No. of family members having these	One	22	100
Being worried excessively or been anxious about several things over the past 6 months	Yes	101	24.0
	No	319	76.0
Worries present most days	Yes	61	60.4
	No	40	39.6
Difficult to control the worries	Yes	33	32.7
	No	68	67.3
The worry interferes with the ability to focus on what are doing	Yes	22	21.8
	No	79	78.2
Other family member having these	Yes	55	13.1
	No	365	86.9
No. of family members having these	One	50	90.9
	Two	2	3.6
	Three	3	5.5

### Depressive Symptoms and Suicide Characteristics

Of the total respondents, 39 (9.3%) reported they have been consistently depressed, hopeless most of the day, nearly every day, for the past 2 weeks or more. About 31 (7.4%) participants could identify another family member who was tearful, hopeless, and complaining about emptiness for the past 2 weeks or more. About 40 (9.5%) of the respondents were less interested in most things or much less able to enjoy the things they were used to enjoy most of the time in the past 2 weeks or more. Additionally, 23 (5.5%) of the participants reported repeatedly wishing to be dead, and 5 (1.2%) attempted suicide. Overall, 55 (13.1%) of the respondents had depressive symptoms (Table 3).

**Table 3:** Depression and suicidal behavior of the respondents and their family at Jimma Zone, Seka Chekorsa district, Southwest Ethiopia, March 2020.

**Variable**	**Characteristics**	**Frequency**	**Percentage**
Consistently depressed or down, hopples most of the day, nearly every day, for the past 2 weeks	Yes	39	9.3
	No	381	90.7
Other family members who were tearful, hopeless, complaining emptiness for the past 2 weeks	Yes	31	7.4
	No	389	92.6
No. of family members having this	one	25	80.7
	Two	4	12.9
	Three	2	6.4
In the past 2 weeks, have you been much less interested in most things or much less able to enjoy the things you used to enjoy most of the time?	Yes	40	9.5
	No	380	90.5
In the past 2 weeks, other family members were less interested in most things or much less able to enjoy the things they used to enjoy most of the time	Yes	28	6.7
	No	392	93.6
No. of family members having this	One	23	82.1
	Two	4	14.3
	Three	1	3.6
In the past month repeatedly consider hurting self	Yes	17	4.0
	No	403	96.0
In the past month have a plan to kill self	Yes	28	6.7
	No	392	93.3
In the past month repeatedly wish dead	Yes	23	5.5
	No	397	94.5
Lifetime suicide attempt	Yes	5	1.2
	No	415	98.8
The family member having a suicide attempt	Yes	12	2.9
	No	408	97.1

### Manic Symptoms

Among the total respondents, 11 (2.6%) had a lifetime period where they feel “up” or “high” or “hyper” or so full of energy or full of self that got into trouble, or other people thought they were not their usual self for 1 week or more. About 38 (9.0%) of the participants described persistently feeling irritable, had arguments or verbal or physical fights, or shouted at people for several days. Overall, 44 (10.5%) of the respondents were identified to have manic symptoms (Table 4).

**Table 4:** Manic symptoms characteristics of the respondents and their family at Jimma Zone, Seka Chekorsa district, Southwest Ethiopia, March 2020.

**Variable**	**Characteristics**	**Frequency**	**Percentage**
Had a period feeling “up” or “high” or “hyper” or so full of energy or full of self that got into trouble, or that other people thought you were not your usual self for at least 1 week	Yes	11	2.6
	No	409	97.4
Other family members having these	Yes	39	9.3
	No	381	90.7
No. of family members having this	One	33	84.6
	Two	5	12.8
	Three	1	2.6
Ever been persistently irritable, for several days, had arguments or verbal or physical fights, or shouted at people	Yes	38	9.0
	No	382	91.0
Other family member having these	Yes	44	10.5
	No	376	89.5
Have you or others noticed that you have been more irritable or overreacted, compared to other people, even in situations that you felt were justified	Yes	53	12.6
	No	377	89.8
Other family member having these	Yes	43	10.2
	No	377	89.8
No. of family members having this	One	32	74.4
	Two	11	25.5

### Respondents' Psychological and Behavioral Characteristics

Among the respondents, 48 (11.4%) had a recent loss of a close family member. About 45 (10.7%) participants were reported being discriminated against. About 12 (2.9%) had confided about being sexually abused, and 118 (28.1%) described financial problems (Table 5).

**Table 5:** Respondents psychological and behavioral characteristics at Jimma Zone, Seka Chekorsa district Southwest Ethiopia, March 2020.

**Variables**	**Characteristics**	**Frequency**	**Percentage**
Any close family member who died recently and feel so bad	Yes	48	11.4
	No	372	88.6
Having any known medical illness	Yes	10	2.4
	No	410	97.6
Currently taking any medication/drug	Yes	7	1.7
	No	413	98.3
Migrated to another country	Yes	10	2.4
	No	410	97.6
Displaced from your home village	Yes	4	1.0
	No	416	99.0
Perceived economic status	Rich	14	3.3
	Medium	114	27.1
	Poor	292	69.5
Experience domestic violence with physical punishment	Present	18	4.3
	Absent	402	95.7
Experience difficulties with family relationships (parents and siblings)	Present	45	10.7
	Absent	375	89.3
Had low emotional support at home during childhood	Present	47	10.7
	Absent	373	88.8
Parent's abuse substance	Present	86	20.5
	Absent	334	79.5
Parents' divorce	Present	39	9.3
	Absent	381	90.7
Family financial problems	Present	118	28.1
	Absent	302	71.9
The low performance or school dropout	Present	113	26.9
	Absent	307	73.1
Perceived discrimination and racism experience	Present	45	10.7
	Absent	375	89.3
Suffered aggression and physical violence	Present	26	6.2
	Absent	394	93.8
Sexual abuse experience	Present	12	2.9
	Absent	408	97.1
Stressful events (relationship problems, separations, and unemployment)	Present	46	11.0
	Absent	374	89.0
Legal problems involvement	Present	11	2.6
	Absent	409	97.4
High frequency of exposition to community violence, theft, assault, and firearms uses	Present	4	1.0
	Absent	416	99.0
Access to mental health service	Present	19	4.5
	Absent	401	95.5
Current pregnancy	Present	14	3.3
	Absent	406	96.7
Currently in the post-partum period	Present	35	3.8
	Absent	385	91.7
Grow by caregiver/other than family	Present	21	5.0
	Absent	399	95.0
Physical and sexual abuse during childhood	Present	6	1.4
	Absent	414	98.6
Living arrangement	Alone	5	1.2
	With immediate family	141	33.6
	With extended family	270	64.3
	With friends	4	1.0

### Factors Associated With Anxiety Symptoms

Male participants had a 50% less risk of developing anxiety symptoms [adjusted OR (AOR), 0.50; 95% CI, 0.29–0.87]. The analysis results also showed rural residents were found to have a 75% less risk of having anxiety (AOR, 0.25; 95% CI, 0.14–0.44). The odds of having anxiety symptoms were ~2 times higher among those who were on the first birth order than those on the third or more birth order. Those employed had 57% less risk of developing anxiety symptoms compared to the housewife counterpart (AOR, 0.43; 95% CI, 0.20–0.90). Respondents whose parents abuse substances had nearly twice increased odds of developing anxiety symptoms (AOR, 2.18; 95% CI, 1.12–4.22). The odds of developing anxiety symptoms were ~5 times higher among those who had perceived discrimination and racism experience (AOR, 5.02; 95% CI, 1.90–13.26). Respondents who had reported stressful event had nearly 4 times' increase of having anxiety symptoms (AOR, 3.96; 95% CI, 1.53–10.24) (Table 6).

**Table 6:** Factors associated with anxiety symptoms of the respondents at Jimma Zone, Seka Chekorsa district, Southwest Ethiopia, March 2020.

**Variables**	**Category**	**Anxiety symptoms**		**COR (95 CI)**	**AOR (95% CI)**
		**No**	**Yes**		
Sex	Male	122 (64.2)	68 (35.8)	0.53 (0.36–0.79)	0.50 (0.29–0.87)^*^
	Female	113 (49.1)	117 (50.9)	1	1
Residence	Rural	117 (70.1)	50 (29.9)	0.37 (0.24–0.56)	0.25 (0.14–0.44)^*^
	Urban	118 (46.6)	135 (53.4)	1	1
Birth order	First	93 (50.8)	90 (49.2)	1.59 (1.01–2.50)	2.20 (1.26–3.84)^*^
	Second	58 (56.9)	44 (43.1)	1.24 (0.74–2.11)	1.32 (0.70–24.8)
	Third or more	84 (62.2)	51 (37.8)	1	1
Occupational status	Farmer	116 (53.0)	103 (47.0)	0.69 (0.43–1.12)	0.64 (0.32–1.25)
	Employed	75 (74.3)	26 (25.7)	0.27 (0.15–0.49)	0.43 (0.20–0.90)^*^
	Housewife	44 (44.0)	56 (56.0)	1	1
Parents abuse substance	Yes	33 (38.4)	53 (61.6)	2.45 (1.51–3.99)	2.18 (1.12–4.22)^*^
	No	202 (60.5)	132 (39.5)	1	1
Perceived discrimination and racism experience	Present	9 (20.0)	36 (80.0)	6.06 (2.84–12.96)	5.02 (1.90–13.26)^*^
	Absent	226 (60.3)	149 (39.7)	1	1
Stressful events^†^	Present	14 (30.4)	32 (69.6)	3.30 (1.70–6.39)	3.96 (1.53–10.24)^*^
	Absent	221 (59.1)	153 (40.9)	1	1

* *Variables significant at P < 0.05*.

† *Stressful events include relationship problems, separations, and unemployment*.

### Factors Associated With Depressive Symptoms

The analysis result has shown depressive symptoms were three and a half times higher among respondents who were unable to read and write compared to primary school and above academic status (AOR, 3.5; 95% CI, 1.3–8.9). Respondents who were on the first birth order were nearly three and a half times more likely to have depressive symptoms compared to those on the third and above birth order (AOR, 3.6; 95% CI, 1.4–8.7). The study participants who had recent bereavement were nearly 4 times more likely to report depressive symptoms (AOR, 3.9; 95% CI, 1.4–10.4). Furthermore, respondents who had depressive symptoms were ~5 times higher to have stressful events compared to their counterparts (AOR, 4.8; 95% CI, 1.7–13.5) (Table 7).

**Table 7:** Factors associated with depressive symptoms of the respondents at Jimma Zone, Seka Chekorsa district, Southwest Ethiopia, March 2020.

**Variables**	**Category**	**Depressive symptoms**		**COR (95% CI)**	**AOR (95% CI)**
		**No (%)**	**Yes (%)**		
Educational status	Unable to read and write	142 (84.5)	26 (15.5)	2.2 (1.1–4.4)	3.5 (1.3–8.9)^*^
	Read and write	65 (80.5)	16 (19.8)	2.9 (1.3–6.5)	5.3 (1.9–14.5)
	Primary school and above	158 (92.4)	13 (7.6)	1	1
Birth order	First	150 (82.0)	33 (18.0)	2.4 (1.2–5.1)	3.6 (1.4–8.7)^*^
	Second	91 (89.2)	11 (10.8)	1.3 (0.5–3.2)	1.1 (0.3–3.3)
	Third or more	124 (91.9)	11 (8.1)	1	1
Having a close family member who is died recently and feel so bad	Present	30 (62.5)	18 (37.5)	5.4 (2.7–10.6)	3.9 (1.4–10.4)^*^
	Absent	335 (90.1)	37 (9.9)	1	1
Stressful event^†^	Present	28(60.9)	18 (39.1)	5.8 (2.9–11.5)	4.8 (1.7–13.5)^*^
	Absent	337 (90.1)	37 (9.9)	1	

* *Variables significant at P < 0.05*.

† *Stressful events include relationship problems, separations, and unemployment*.

### Factors Associated With Manic Symptoms

Respondents who had depressive symptoms were almost four and a half times more likely to have manic symptoms (AOR, 4.3; 95% CI, 1.71–11.02). Study participants with parental substance use history were 3 times more at risk of having manic symptoms (AOR, 2.8; 95% CI, 1.21–6.69). The result of the regression analysis has revealed respondents who had perceived discrimination and racism experience had 5 times more likely to report manic symptoms (AOR, 5.0; 95% CI, 1.96–12.77). Firstborn children had ~4 times higher risk of developing manic symptoms compared to those on the third or more birth order (AOR, 4.1; 95% CI, 1.29–12.98). Study participants who perceived their economic status as a medium were two and a half times more likely to have manic symptoms than those who perceived low economic status (AOR, 2.3; 95% CI, 1.06–5.24) (Table 8).

**Table 8:** Factors associated with manic symptoms of the respondents at Jimma Zone Seka Chekorsa district Southwest Ethiopia, March 2020.

**Variables**	**Category**	**Manic symptoms**		**COR (95 CI)**	**AOR (95% CI)**
		**No**	**Yes**		
Depressive symptoms	Yes	37 (67.3)	18 (32.7)	1	1
	No	339 (92.9)	26 (7.1)	6.3 (3.18–12.64)	4.3 (1.71–11.02)^*^
Parents abuse substance	Present	62 (72.1)	24 (27.9)	6.0 (3.16–11.67)	2.8 (1.21–6.69)^*^
	Absent	314 (94.0)	20 (6.0)	1	1
Perceived discrimination and racism experience	Present	28 (62.2)	17 (37.8)	7.8 (3.81–16.05)	5.0 (1.96–12.77)^*^
	Absent	348 (92.8)	27 (7.2)	1	1
Birth order	First	159 (86.9)	24 (13.1)	3.2 (1.28–8.17)	4.1 (1.29–12.98)^*^
	Second	88 (86.3)	14 (13.7)	3.4 (1.26–9.24)	4.7 (1.37–16.64)^*^
	Third or more	129 (95.6)	6 (4.4)	1	1
Perceived economic status	Medium	107 (83.6)	21 (16.4)	2.2 (1.21–4.32)	2.3 (1.06–5.24)^*^
	poor	269 (92.1)	23 (7.9)	1	1

* *Variables significant at P < 0.05*.

## Discussion

The study aimed to assess the magnitude and factors associated with anxiety, depressive, and manic symptoms, and suicidal behavior among the Jimma zone community.

The finding of this study showed 55 (13.1%) of the respondents had depressive symptoms. Consistent findings were reported from the pooled prevalence of depression in Ethiopia (51), rural communities in Malaysia (11.30%) (10), Brazil (14%) (52), northwest Ethiopia (17.5%) (53), and Puerto Rico (17.8%) (54). The findings in all studies have shown that depression is a global problem. However, this study finding is higher than the 4.4% WHO global and regional estimates of the prevalence of depression in 2015 (55). The current study was conducted 5 years later than the WHO study; various changes such as economic inflation, ethnic violence, increased Khat (local stimulant leaf), and other substance use in the area could be the probable contributing factors for the high prevalence of depression in the area. Yet, the result is lower than the studies done among adolescents living in the cities of Kenya (26.4%) (56) and South Brazil (35.4%) (11). The discrepancy could be explained by as follows: this study was conducted in the rural area of the country compared to the Kenya and Brazil studies. Concentrations of low socioeconomic status; low social capital, e.g., social support; and higher rates of pollution [e.g., air, water, and noise pollution in the cities might be the reason for the high prevalence of depression symptoms (57, 58)].

Manic symptoms were noted in 44 (10.5%) of the respondents; similarly, the US National Epidemiologic Catchment Area database showed that the prevalence of subthreshold bipolar symptoms was 5.1% (59). At present, no data exist that indicate how many patients there are with such subthreshold bipolar symptoms in the developing countries and community setting. The high prevalence of substance (Khat) use, socioeconomic pressure, and limited availability of mental health treatment centers in the area might contribute to the symptoms (39, 60).

The finding of this study has shown 185 (44.0%) of the respondents had anxiety symptoms. This finding is higher than those found in the studies done in Malaysia (8.2%) (15), from African cultures (7.3%) to Euro/Anglo cultures (10.4%) (61), rural communities of Northern India (22.7%) (62), and Kashmir valley (26%) (17). The worries and uncertainty resulting from living in poverty seem to be an important driver of mental health problems including anxiety, as do the effects of low income on childhood development and one's living environment (63).

In this study, 5.5 and 1.2% of the participants reported repeatedly wishing dead and attempting suicide, respectively. A consistent finding was reported from the study done in Addis Ababa, Ethiopia, in which the rates of suicidal ideation and attempt were 2.7 and 0.9%, respectively (64). Another population study in Ethiopia has shown 13.5% of the study participants had suicidal ideation, and 1.8% had suicide attempt (65). Additionally, a study was done in Nigeria that revealed 7.28% had suicidal ideation (66). A study done in rural communities in China reported 4.8% and 0.4% had suicidal ideation and attempt, respectively (67). However, our finding was lower than the findings of 17.1% for suicidal ideation and 2.8% for suicide attempts from Brazil (68). The discrepancy could be explained by the differences in the social control networks, extended family ties, religious, cultural practices differences, and different stressors in the study settings (7, 69).

The finding of this study has revealed that respondents with higher educational status had less risk of developing depression. This is consistent with the studies done in Iran (18) and Pakistan (70). This could be because education as a means enables people to gain success in life and may fundamentally contribute to the emotional well-being of the person (71). In the study area, an individual with higher education status usually has better work opportunities, health information, and living standards. This could protect them against developing depression (72). Nevertheless, individuals with higher education backgrounds were comparatively more prone to mood disorders in the study done in the United States (10). This might be explained in that in Western culture highly educated people seek well-paid jobs and may have better socioeconomic expectations. In their race to meet these expectations, they could experience unmanageable stress, which predisposes them to mood disorders (73).

In the current study, respondents with first and second birth order status were more prone to have depression symptoms than those with third and above birth order status. This finding is in line with the study done in Nepal (74). However, no difference was observed in the study done in Egypt (75). Similarly, birth order was found to have an association with anxiety symptoms in this study. This finding was consistent with the study done in Kuwait (69). Birth order is one of the most significant life factors, and it is the best indicator of the kind of personality someone has. In the study area, the oldest child commonly has many responsibilities compared to the youngest child of the family. Studies also have shown that because of much expectations that are placed on the oldest child in a family, the eldest one experiences more emotional disturbance and struggles in coping with the stressful condition (76). In addition, the firstborns were considered as the smaller version of their parents; therefore, they have received much more control and attention from their parents. Hence, they tend to be over responsible, reliable, well-behaved, and careful. This might explain a higher level of emotional disturbances in this group (77).

In our study, respondents with self-perception of their economic status as a medium were at higher risk of having manic symptoms. In many studies, it was usually noted that bipolar disorder was relatively higher among those who have medium socioeconomic status than that of controls or the general population (78, 79). However, many current studies have failed to confirm such assumptions (12). The anticipation of economic shocks may cause mental illness such as mania. People living in poverty face substantial uncertainty and income volatility and complex financial portfolios, often without access to formal insurance; this might increase the risk of developing bipolar symptoms (80, 81).

In the current study, having depressive symptoms was associated with an increased risk of manic symptoms. This might be explained by bipolar disorders beginning with depressive episodes, and a significant proportion of individuals who had initial major depressive disorder will later be reclassified as having a bipolar disorder (82). Various precipitating factors, such as socioeconomic stress, poor control of depressive-manic symptoms through medication, and the nature of the illness by itself, might be the reason for manic-depressive cycle (83, 84).

Perception of prejudice and discrimination based on ethnicity increased the risk of presentation of manic and anxiety symptoms among the respondents. Stress associated with the experiences of perceived racial discrimination and prejudice has substantial negative effects on both physical and mental health and might precipitate mental health problems (85). Discrimination may contribute to psychological problems through numerous possible mechanisms including negative psychological and physical stress response, hypervigilance, and increased involvement in unhealthy behaviors (20, 86, 87). In Ethiopia, ethnicity-related violence in different parts of the country could be the reason for the death, and internal displacement of people from their living area can be considered as a serious stressor for manic symptoms eruption (88, 89).

In this study, respondents whose parents abuse substances were more likely to have anxiety symptoms. This could be because an individual who had parents with abusing substances were having problems including poor attachments, economic difficulties, legal problems, emotional abuses, and violence (90).

Most the studies conducted on depression, anxiety, mania, and suicide behavior in Ethiopia were among prisoners (91), substance users (92), university staffs and students (93, 94), women (95), pregnant women (96, 97), postpartum mothers (98), children (99), medically ill patients (100–103), epileptic patients (104), disorder threshold level (39, 105, 106), and in the context of common mental disorders (13, 107). Our study finding has a unique contribution as it has revealed the rural community mental health problems characteristics.

However, this article has presented psychiatric symptoms, not disorders. Additionally, some of the discussion comparisons were made with many countries that are culturally different from this study setting. So, the generalization and conclusions should be made cautiously. Moreover, as it is cross-sectional by nature, it does not show the cause-and-effect relationship between the outcome and explanatory variables. Ethiopia has registered the first COVID-19 cases on March 13, 2020, the period where this study was conducted. Even though the study was conducted in a rural part of Ethiopia, where the spread of the infection was gradual, this might affect the results of this study. The current study did not assess some of the anxiety symptoms in agoraphobia, posttraumatic stress disorder, and obsessive–compulsive disorder. Furthermore, the study tool to assess the variables of this study was not validated in the local, Afan Oromo, and Amharic languages. Additionally, the latest version of the M.I.N.I. questionnaire was not used because the study tool was restricted by the publisher for free use.

### Future Directions

Based on this study's limitations, we recommend interested researchers in the field of mental health and public health to investigate further the magnitude of psychiatric disorders with the latest and validated tools in local languages with a larger sample size. Furthermore, longitudinal studies are recommended to explore the cause-and-effect association of the outcome and explanatory variables.

### Clinical Implications of the Study

This study may have an immense contribution in improving the mental health service of the study area by revealing the magnitude of the problems and contributing factors. In the study setting, there were very limited mental health services, which do not match with the rates of mental health problems as found in this study. This study will further motivate the researchers to evaluate the study population's intention to use mental health services, and the presence of stigma on patients with mental health problems and services use that require effective locally sound education programs in the study setting.

## Conclusions

The study has revealed that a significant proportion of the community members have anxiety depressive, manic symptoms, and suicidal behavior. Furthermore, various risk factors were identified to have an association with the problems. Therefore, appropriate community-based mental health services should be designed and implemented to address the negative impact of the problems.

## Data Availability Statement

The original contributions presented in the study are included in the article/supplementary material, further inquiries can be directed to the corresponding author/s.

## Ethics Statement

The studies involving human participants were reviewed and approved by the institutional review board of Jimma University, Institute of health. The patients/participants provided their written informed consent to participate in this study.

## Author Contributions

YT was the principal investigator of the study and was involved from inception to design acquisition of data, analysis, and interpretation, and drafting and editing of the manuscript. LA, SA, GT, ZB, GA, MG, and KY were involved in the reviewing of the proposal, tool evaluation, interpretation, and critical review of the draft manuscript. All authors read and approved the final manuscript.

## Conflict of Interest

The authors declare that the research was conducted in the absence of any commercial or financial relationships that could be construed as a potential conflict of interest.

## Abbreviations

AOR: Adjusted Odds Ratio
CI: Confidence Interval
CORm: Crude Odds Ratio
OR: Odds Ratio
SD: Standard Deviation
SPSS: Statistical Package for the Social Sciences
US: United State.
